# Intrafraction cone beam computed tomography verification of breath hold during liver stereotactic radiation therapy

**DOI:** 10.1002/jmrs.441

**Published:** 2020-10-06

**Authors:** Elizabeth Brown, Erika Muscat, Patrick O’Connor, Howard Liu, Yoo‐Young Lee, David Pryor

**Affiliations:** ^1^ Radiation Oncology Princess Alexandra Hospital Brisbane Queensland Australia; ^2^ School of Clinical Sciences Queensland University of Technology Brisbane Queensland Australia; ^3^ Radiation Oncology Department Sunshine Coast University Hospital Adem Crosby Centre, Birtinya Queensland Australia; ^4^ School of Medicine University of Queensland Brisbane Queensland Australia

## Abstract

**Introduction:**

Intrafraction imaging is an Elekta feature that enables cone beam computed tomography (CBCT) acquisition simultaneously with treatment arc delivery. It has facilitated the introduction of breath‐hold (BH) gated stereotactic body radiation therapy (SBRT) by enabling visualisation of tumour and organs at risk during treatment. The aims of this study were to assess BH reproducibility and use intrafraction CBCT (IF‐CBCT) to quantify any variation in diaphragm position (diaphragmatic feathering) during the multiple BHs performed during each arc.

**Methods:**

IF‐CBCTs for consecutive liver SBRT patients where BH was achieved using the Elekta Active Breathing Control (ABC) system were retrospectively evaluated. Average intrafraction couch shifts for deep‐inspiration BH (DIBH) or end‐expiration BH (EEBH) were recorded as an indication of reproducibility. Diaphragmatic feathering was quantified by measuring the difference between the most superior and inferior visible edges of the diaphragm on IF‐CBCTs.

**Results:**

A total of 212 images from 30 patients were reviewed. Twenty‐two (73.3%) patients were treated in EEBH. The mean intrafraction shift was similar between DIBH and EEBH groups with the largest mean shift of 0.22cm occurring in the superior–inferior direction. Mean diaphragmatic feathering was similar between the DIBH and EEBH groups, 0.09cm (0‐0.44cm) and 0.14cm (0–1.89cm) respectively. A higher percentage of EEBH patients demonstrated no diaphragmatic feathering throughout treatment compared with DIBH patients (31.8% vs 25%).

**Conclusion:**

The results of this study indicate that BH is reproducible in both DIBH and EEBH for liver SBRT treatment using the ABC system. Appropriate patient selection and BH coaching prior to CT simulation are critical to its success.

## Graphical abstract

Intrafraction cone beam computed tomography (IF‐CBCT) has confirmed the reproducibility of both end‐exhalation breath hold (EEBH) and deep‐inspiration breath hold (DIBH) using Active Breathing Control during liver stereotactic body radiation therapy (SBRT). Intrafraction breath hold (BH) variability can be quantified online and soft tissue anatomy directly visualised which can inform individualised adaptation of treatment delivery. Appropriate patient selection and BH coaching prior to CT simulation remain critical to its success.

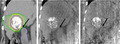

## Introduction

Stereotactic body radiation therapy (SBRT) is an effective treatment modality for patients with primary or oligometastatic cancer in the liver.^1^, ^2^, ^3^ Studies have reported high rates of local control and low rates of significant treatment related toxicity.^1^, ^4^ However, liver tumours are highly susceptible to respiratory‐induced motion with reports of movement in the range of 5 to 50 mm craniocaudally during free breathing.^5^, ^6^ Strategies to optimise motion management are therefore critical. A number of approaches have been utilised to reduce the impact of motion including abdominal compression, gating, planning on mid‐ventilation phase and breath hold (BH) including assisted techniques such as the Elekta Active Breathing Coordinator (ABC) system (Elekta Oncology, Crawley, United Kingdom). Reports have demonstrated the feasibility, accuracy and reproducibility of the ABC system in liver SBRT patients.^5^, ^7^, ^8^ However, there has been a lack of published literature on the use of volumetric imaging to assess intrafraction variation over the duration of a BH treatment, or the degree of motion blur artefact visualised on imaging caused by multiple unique BHs.

**Figure 1:**
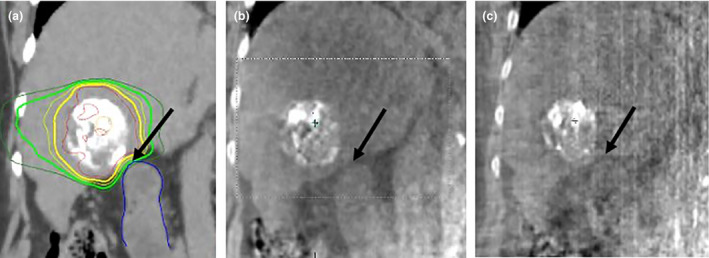
Planning CT (a) showing intra‐tumoural lipiodol with black arrow at region of steep dose gradient at interface between tumour and duodenum (blue contour). Adequate visualisation of tumour–duodenum interface is maintained with pre‐treatment CBCT (b) and intrafraction volumetric imaging (IF‐CBCT) (c) allowing accurate placement of steep dose gradient

Intrafraction imaging, herein referred to as IF‐CBCT, is a licensable feature of Elekta XVI (Elekta Oncology, Crawley, United Kingdom) that enables cone beam computed tomography (CBCT) acquisition simultaneously with megavoltage (MV) treatment field delivery.^9^ This 3D acquisition reconstructs as a CBCT scan immediately after completion of the treatment arc. As such, it allows online volumetric verification of the liver or surrogate marker position in addition to the adjacent organs at risk (OAR) for each completed arc (Fig. 1). This provides confirmation of intrafraction stability and reproducibility of the multiple BHs required during treatment. The aims of this study were 1) to assess BH reproducibility by analysing intrafractional shifts using IF‐CBCT in a cohort of liver SBRT patients and 2) to quantify the potential maximal variability in BH by using the position of the diaphragm as a surrogate.

## Methods

### Patient selection

The first 30 consecutive liver SBRT patients treated in BH using the Elekta ABC system following implementation of IF‐CBCT at our institution in January 2018 were included in this retrospective quality assurance study. Patients were considered suitable for treatment in BH (either end exhalation (EEBH) or deep inspiration (DIBH)) if they could meet the following criteria: 1) be able to follow prompts from staff, 2) be able to establish a reproducible breathing pattern in regular breathing and 3) be able to hold their breath for a minimum of 15 seconds. Additionally, the reproducibility of the tumour position in repeat BH CT simulation images must be of an acceptable level to allow a reduction in planned tumour volume (PTV) margins compared with free breathing PTV margins. The departmental preference is to treat these patients in EEBH where possible with the exception of tumours adjacent to pericardium where DIBH may increase separation. This study received ethics exemption from the Metro South Human Research Ethics Committee as it was a quality improvement study involving no intervention in patient treatment.

### CT simulation and planning

All patients were positioned supine with their arms above their head immobilised in a custom moulded Elekta BodyFIX bag (Elekta Oncology, Crawley, United Kingdom). If required, a support was placed under their knees for comfort. CT simulation was performed with 2mm thick slices on either a Toshiba Aquilion (Canon Medical Systems Corporation, Otawara, Japan) or Phillips Brilliance Big Bore (Koninklijke Philips, Amsterdam, Netherlands) scanner. Planning and contrast enhanced CT scans, all acquired in BH, were performed in one or more of the arterial, venous or delayed phases. These scans were used for target volume delineation and assessment of BH reproducibility. A minimum of two CT scans were required to assess BH reproducibility with most patients having three to four scans acquired. The standard threshold for BH reproducibility was 0.5cm; however, this was assessed on an individual patient basis with the radiation oncologist making the final decision regarding the motion management employed for treatment.

The gross tumour volume (GTV) was defined on the contrast‐enhanced CT along with co‐registration of magnetic resonance imaging (MRI) sequences if available. The PTV was generated using a 0.5cm expansion radially and 0.8cm craniocaudally (minimum 0.5cm) expansion on the GTV to account for setup uncertainty and variable BH position. No internal target volume (ITV) was created as variability in BH reproducibility was accounted for in the GTV to PTV expansion. All patients were planned with two partial 6MV flattening filter free (FFF) volumetric modulated arc therapy (VMAT) arcs appropriate for the tumour location with the isocentre placed in the centre of the PTV.

### Treatment and intrafraction shifts

Patients were positioned as per the instructions from the CT simulation session. The treating radiation oncologist was present for all treatment sessions and approved online registrations for all images acquired. Pre‐treatment CBCT imaging was performed with all shifts applied to accurately position the treatment beam. A second CBCT was performed to confirm shift accuracy. IF‐CBCT imaging was performed on all treatment beams. IF‐CBCT acquisition was paused in conjunction with interruption of the treatment beam when the patient was not in BH as IF‐CBCT acquisition is triggered by gantry movement. Both the CBCT and IF‐CBCT image slices were 2mm thick. All shifts were applied after online IF‐CBCT registration of the first arc. Intrafraction couch shifts for each BH type (DIBH or EEBH) were recorded for all IF‐CBCT images acquired. In general, mid‐treatment shifts were not applied if under the 0.2cm tolerance. Matching priorities varied between patients with some treatments requiring a best fit approach to ensure adjacent OARs did not exceed tolerance. Treatment‐related factors including BH duration, number of BH required and treatment time were also recorded for all patients.

### Assessment of intrafraction BH‐induced motion blur

**Figure 2:**
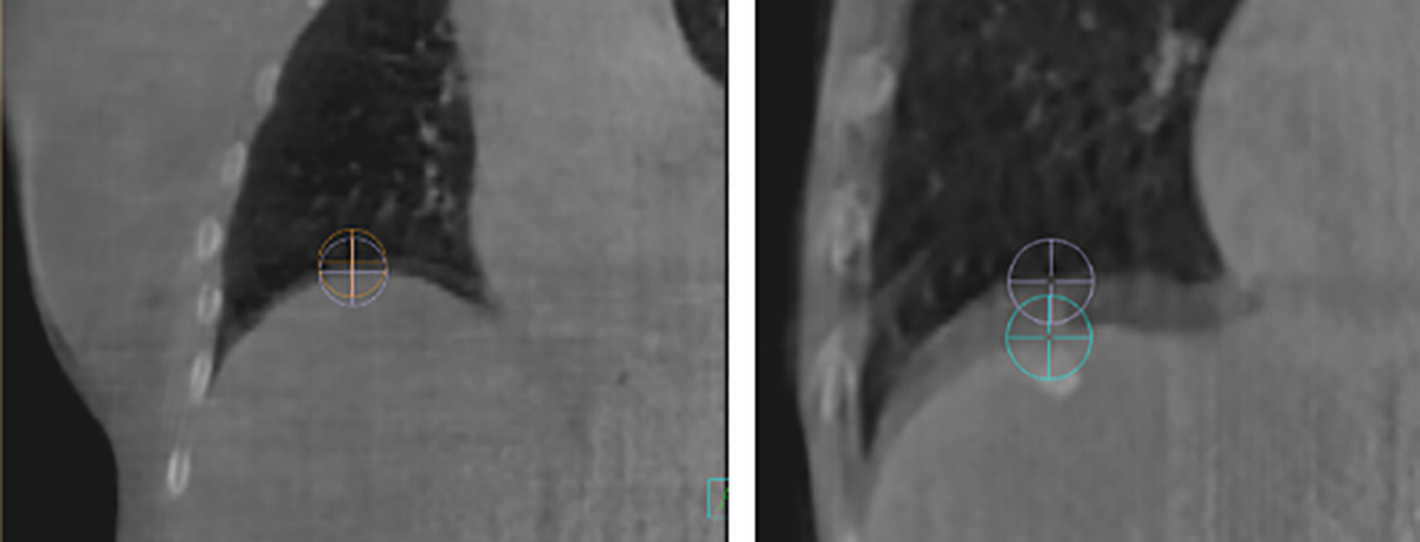
Example of BH‐induced motion blur artefact on the IF‐CBCTs of two different patients. The purple and blue points demonstrate the most superior and inferior visible edges of the diaphragm

Slight changes in anatomical position can result in small amounts of blurring artefact in the images acquired. This can give the diaphragm a ‘feathered’ appearance once the IF‐CBCT is reconstructed (Fig. 2). IF‐CBCT image frames are acquired when the patient is in BH; therefore, IF‐CBCT acquisition is comprised of multiple BHs. This means that any variation in BH position contributes to the diaphragmatic feathering visualised in the final IF‐CBCT. Measurement of diaphragmatic feathering provides an indication of BH reproducibility during each treatment arc.

The extent of BH‐induced diaphragmatic feathering visible on IF‐CBCT was defined as the superior–inferior distance between the visible edges of the diaphragm at the diaphragm–lung interface on the coronal slice (Fig. 2). Firstly, the IF‐CBCT was registered with the planning CT in Pinnacle v16 (Koninklijke Philips, Amsterdam, Netherlands) by two experienced radiation therapists. Images were registered by matching the isocentre position on both image sets. The registration parameters were not further adjusted to replicate the couch shifts applied during treatment to correct for daily target volume motion as this would change the spatial relationship between diaphragm position and isocentre position. Two points were placed on the visible superior edges of the diaphragm in the coronal view with imaging visualised using the standard lung window level. The same anterior–posterior location was used for both points and was determined by finding the most superior point of the diaphragm on the sagittal slice of the planning CT. The difference between the superior–inferior position of these two points was recorded (Fig. 2). A random check of point placement accuracy was performed on 25% of the matches by another radiation therapist. Not all tumours were near the diaphragm, so this metric was not a true reflection of the variability of target position for these treatments. It was, however, a readily definable surrogate.

### Statistical analysis

Descriptive statistics were used to evaluate the cohort as a whole as well as to compare patients treated in DIBH with those treated in EEBH. Mann–Whitney tests were used to compare treatment‐related parameters in addition to IF‐CBCT shifts and diaphragmatic feathering results of the DIBH and EEBH patient groups. Spearman’s rank correlation was used to test for any potential associations between magnitude of diaphragmatic feathering and treatment parameters. These tests were used as the data were not normally distributed. A p‐value of < 0.05 was considered statistically significant. Data were analysed using the Stata (version 12.1, StataCorp LP, Texas, USA) program.

## Results

Between January and October 2018, the first 30 liver SBRT patients treated in BH using the Elekta ABC system with IF‐CBCT were included in this retrospective analysis. Twenty (66.7%) were male, and the median age was 68 years (range 51–85). Eighteen (60%) patients were treated with a 5‐fraction schedule (dose range 30–50 Gy) whilst 12 (40%) were treated with a 3‐fraction schedule (dose range 30 to 50 Gy). Significantly more patients were treated in EEBH (73.3%, p = 0.02). The primary reasons for using DIBH were to increase the separation of superomedial tumours from pericardium or for patients unable to reproducibly sustain EEBH for 15 seconds. Treatment parameters are displayed in Table 1. Other treatment parameters were comparable between the groups with only a marginally higher mean number of BHs required per fraction in the EEBH cohort.

**Table 1:** Treatment parameters

Parameter	DIBH (*n* = 8)	EEBH (*n* = 22)	p‐value
Mean BH duration (s) (range)	23.3 (18–28)	24.1 (19–25)	0.55
Mean number of BH per fraction (range)	9.8 (7–13)	11.2 (8–13)	0.06
Mean treatment time (mins) (range)	14.9 (12–18)	14.6 (8–25)	0.46

BH, breath hold; DIBH, deep‐inspiration breath hold; EEBH, end‐exhalation breath hold.

### IF‐CBCT shifts

A mean of seven (range 2–10) IF‐CBCT images were analysed for each patient. The wide range in the number of IF‐CBCT images acquired was a result of differences in prescribed fractions and some missed IF‐CBCT images due to errors occurring during acquisition. A total of 34 images were missed due to an unpredictable trigger pulse fault that was fixed in later software versions. Details of the IF‐CBCT shifts are found in Table 2. A smaller percentage of IF‐CBCT images had a shift applied in the EEBH patient group; however, this was not significant (17.9% vs 22.9%). The mean magnitude of the variance between the reference and IF‐CBCT images when greater than 0 was similar between the DIBH and EEBH groups with the largest mean shift of 0.22cm occurring in the superior–inferior direction. Likewise, the highest percentage of IF‐CBCT shifts greater than or equal to the imaging tolerance of 0.2cm were in the superior–inferior direction followed by anterior–posterior. When stratifying these results into BH type, the percentage of IF‐CBCT shifts ≥ 0.2cm was also highest in the superior–inferior direction for both groups with this accounting for over 50% of shifts.

**Table 2:** IF‐CBCT shifts

Parameter	DIBH (n = 8)	EEBH (n = 22)	p‐value
Number of images	48	156	
Number of images shifted (≥ 0.2 cm)	11 (22.9%)	28 (17.9%)	0.36
Mean Sup‐Inf shift (cm) (range)	0.2 (0–0.3)	0.22 (0–0.6)	0.66
Mean Left‐Right shift (cm) (range)	0.15 (0–0.2)	0.16 (0–0.2)	0.84
Mean Ant‐Post shift (cm) (range)	0.18 (0–0.2)	0.16 (0–0.4)	0.38
Number of IF‐CBCT shifts ≥ 0.2cm Sup‐Inf Left‐Right Ant‐Post	DIBH and EEBH combined 31 (33.4%) 10 (17.2%) 17 (29.3%)		

Ant, anterior; DIBH, deep‐inspiration breath hold; EEBH, end‐exhalation breath hold; IF‐CBCT, Intrafraction cone beam computed tomography; Inf, inferior; Post, posterior; SD, standard deviation; Sup, superior.

### Intrafraction BH‐induced diaphragmatic feathering

The results of the diaphragmatic feathering assessment can be seen in Table 3. One image was excluded from the DIBH group as the superior portion of the diaphragm was not visible.

**Table 3:** Diaphragmatic feathering results

Parameter	DIBH (n = 8)	EEBH (n = 22)	p‐value
Number of images	47	156	
Mean feathering (cm) (range)	0.09 (0–0.44)	0.14 (0–1.89)	0.41
Number of images with feathering ≥ 0.2 cm	12 (25.5%)	46 (29.5%)	0.60
Number of images with feathering ≥ 0.5 cm	0	15 (9.6%)	0.09
Number of patients with no feathering	2 (25%)	7 (31.8%)	0.72

DIBH, deep‐inspiration breath hold; EEBH, end‐exhalation breath hold.

DIBH and EEBH groups demonstrated mean feathering of 0.09cm and 0.14cm, respectively, with the proportion of images demonstrating diaphragmatic feathering ≥ 0.2cm comparable between the two groups (p = 0.60). Of interest is the wide range of feathering results recorded in the EEBH group compared with DIBH with maximum feathering of 1.89cm and 0.44cm respectively. This was further demonstrated by the greater number of images in the EEBH group with feathering ≥ 0.5cm. Despite this wide range of results, a higher percentage of EEBH patients demonstrated no diaphragmatic feathering throughout their treatment.

**Figure 3:**
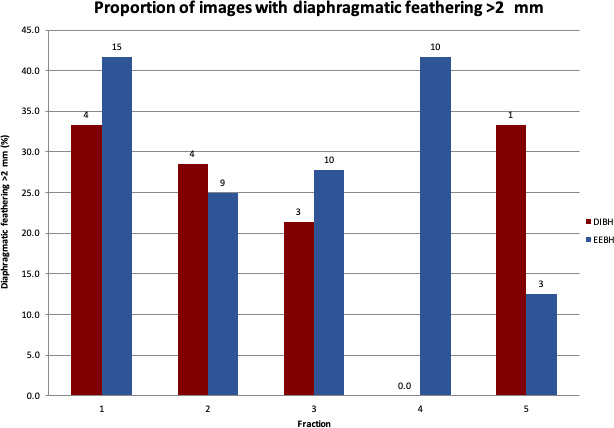
Proportion of images with diaphragmatic feathering> 0.2cm. Figures above the bars indicate the absolute number of patients. DIBH – deep‐inspiration breath hold, EEBH – end‐exhalation breath hold

In reviewing the proportion of images with diaphragmatic feathering> 0.2cm over the course of treatment, the percentage decreased as treatment progressed (Fig. 3). At fraction 1, more EEBH patients displayed diaphragmatic feathering than DIBH patients (41.7% vs 33.3%); however, this became more comparable as treatment progressed with 27.8% of EEBH and 21.4% of DIBH patients demonstrating> 0.2cm feathering at fraction 3. It is important to highlight the considerable decrease in IF‐CBCT images assessed after fraction 3 due to only 40% of patients prescribed> 3 fractions. The number of IF‐CBCT images assessed for the DIBH group decreased by 71.5% and the EEBH group decreased by 33.3%.

**Figure 4:**
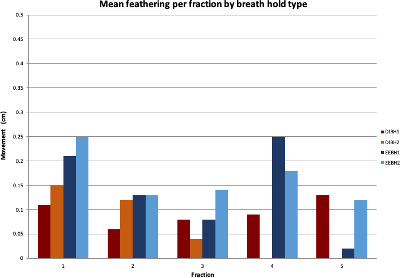
Mean diaphragmatic feathering for each arc per fraction by breath hold type. DIBH – deep‐inspiration breath hold, EEBH – end‐exhalation breath hold, 1‐ arc 1, 2 – arc 2

When dissected into the magnitude of feathering per arc, generally more feathering was observed in the second arc for fractions 1 to 3 in both groups; however, this was not statistically significant (p = 0.17) (Fig. 4).

### Treatment parameter associations

No significant associations were found between the magnitude or incidence of diaphragmatic feathering and the following treatment parameters: number of BH, treatment time, number of CBCTs and difference in arc monitor units.

## Discussion

The implementation of IF‐CBCT has facilitated direct soft tissue visualisation of the liver (or associated tumour surrogate) in addition to adjacent critical OARs during SBRT. This allows confirmation of accuracy during treatment and gives the ability to identify and quantify intrafraction variability. We found a high level of reproducibility using the ABC system in both EEBH and DIBH. Less than 20% of images had an intrafraction shift applied with this shift being 0.2cm on average when it was required.

This is the first article to the best of the author’s knowledge utilising IF‐CBCT to assess diaphragmatic feathering in liver SBRT patients. We looked at diaphragmatic feathering at the apex of the diaphragm to quantify the largest potential intrafraction variability per treatment arc. These values were higher than the recorded IF‐CBCT shifts as some tumour locations in the liver (e.g. anterior, medial) have less excursion than the apex or posterior aspect of the diaphragm. Overall, the mean diaphragmatic feathering observed was less than 0.2cm with 30% of patients demonstrating no feathering over the course of treatment and < 10% of patients with> 0.5cm.

This is consistent with other studies in the literature investigating diaphragm stability with patients treated in EEBH.^5^, ^10^ EEBH is the preferred method of BH as it has been found to be highly reproducible. Both Eccles et al and Zhong et al found mean movement less than 0.2cm when using kV fluoroscopy.^5^, ^10^ We found no significant differences in intrafraction shifts and diaphragmatic feathering between patients treated in EEBH and DIBH when utilising the ABC system indicating that DIBH is a viable motion management option when patients cannot achieve EEBH.

Some outliers were observed with 9.6% patients having feathering> 0.5cm. Whilst a higher percentage of patients treated in EEBH displayed no diaphragmatic feathering throughout treatment, the EEBH cohort did have a wider range (maximum feathering of 1.89cm vs 0.44cm) dominated by an outlier (1.89cm during the second arc of the first treatment session). As it appeared that this feathering was only present for a portion of one BH during one fraction, its significance was considered negligible. On review of all CBCTs for this patient, including the dummy run session, this was the only instance of diaphragmatic feathering of this magnitude occurring. Re‐coaching was performed after the first treatment session to assist with reproducible BH for the entirety of a treatment fraction.

This highlights the importance of appropriate patient selection, comprehensive BH coaching including dummy runs prior to fraction one and the value of intrafraction verification of BH. Upon review of this limited cohort, no strong pre‐treatment indicators were observed identifying patients who may have problems with BH during treatment. This further emphasises the importance of treatment verification imaging and the need to intervene as appropriate when problems are observed. It is also important that both individual and population‐based breath‐hold reproducibility is assessed at the local level to ensure appropriate PTV margins are employed.^8^ Our departmental protocol currently recommends 0.8cm craniocaudal margins for BH cases.^3^ The degree of BH reproducibility with ABC combined with soft tissue visualisation on CBCT and IF‐CBCT can allow for more reliable placement of steep dose gradients, for example in the case of tumours in close proximity to luminal gastrointestinal structures or pericardium (Fig. 1). Whilst IF‐CBCT does not equate to real‐time tracking, the images can be reviewed online and adaptations made during treatment. Where a reliable surrogate is well visualised on CBCT and IF‐CBCT and where BH is demonstrated to be stable and reproducible, further margin reductions could be employed.

Reviewing diaphragmatic feathering over the course of treatment showed that mean diaphragmatic feathering decreased in magnitude for both BH types as treatment progressed. It is hypothesised that this is a result of patients becoming increasingly comfortable with BH the more times it is performed, again highlighting the potential value of coaching sessions and dummy runs. Of note, the proportion of images with> 0.2cm of diaphragmatic feathering was larger in the EEBH group than the DIBH group at fraction 1 (41.7% vs 33.3%); however, this became more comparable as treatment progressed. Anecdotally staff report that patients are more familiar with the concept of DIBH whereas EEBH can be a more difficult idea to master and hold for 15 seconds or more. This finding raises the question of whether multiple BH coaching sessions prior to CT simulation for patients being treated in EEBH warrant further investigation to enable them to become more comfortable with the technique.

A non‐significant trend towards increased diaphragmatic feathering was seen for the second arc of each treatment session. To reduce the potential impact of fatigue on BH reproducibility, we aim to minimise overall time on the treatment couch by using 2 coplanar FFF VMAT arcs. In this study, the mean treatment time was less than 15 minutes with some treatments as short as 8 minutes. The use of IF‐CBCT also negates the need for additional mid‐treatment CBCTs further shortening overall time.

There were some limitations to this study. The sample size was small as this was a quality improvement study. This may mean that the results are not truly representative of the patient population. Additionally, as this cohort included the first series of liver SBRT patients treated in BH at our institution, it is possible that these data do not represent what is now being achieved as staff have become increasingly familiar and confident with BH liver SBRT, particularly in EEBH. It is also important to note that there are limitations in using the diaphragm as a surrogate marker for all tumours as it is only most useful in cases where the tumour is located near the dome of the diaphragm. For central or peripheral lesions below the dome, utilisation of the liver edge or fiducial markers may be more accurate for treatment delivery.^11^

Future work could include the identification of predictive factors to better select those patients suitable for treatment in BH and the dissection of IF‐CBCT into individual BHs to try to determine the proportion of treatment delivered in each BH and better understand the potential dosimetric impact of differences in BH position. True real‐time tracking with continuous kilovoltage intrafraction monitoring (KIM) of fiducials is also being explored in the ongoing TROG 17.03 (LARK) trial enabling the opportunity for further margin reduction.^12^ Further training of these algorithms for markerless tracking may also allow for non‐invasive real‐time tracking solutions using soft tissue structures such as the diaphragm and contrast agents such as lipiodol.

## Conclusion

IF‐CBCT has confirmed the reproducibility of both EEBH and DIBH using ABC during liver SBRT. Intrafraction BH variability can be quantified online and soft tissue anatomy directly visualised which can inform individualised adaptation of treatment delivery. Appropriate patient selection and BH coaching prior to CT simulation remain critical to its success.
